# Median Arcuate Ligament Syndrome Masquerading as Functional Abdominal Pain Syndrome

**DOI:** 10.7759/cureus.20573

**Published:** 2021-12-21

**Authors:** Michael Scharf, Kaitlyn A Thomas, Niteesh Sundaram, Shri Jai Kirshan Ravi, Mustafa Aman

**Affiliations:** 1 Surgery, Geisinger Commonwealth School of Medicine, Scranton, USA; 2 Clinical Education, Lake Erie College of Osteopathic Medicine, Elmira, USA; 3 Surgery, University of Pittsburgh School of Medicine, Pittsburgh, USA; 4 Gastroenterology, Guthrie Robert Packer Hospital, Sayre, USA; 5 Surgery, Guthrie Robert Packer Hospital, Sayre, USA

**Keywords:** delayed diagnosis, dunbar syndrome, functional abdominal pain syndrome, laparoscopic surgical repair, celiac axis compression

## Abstract

Median arcuate ligament syndrome refers to anatomical compression of the celiac artery and/or ganglion by fibrous attachments of the median arcuate ligament. It typically presents as a vague constellation of abdominal symptoms that are often initially attributed to various other gastrointestinal pathologies; thus, it can be very difficult to diagnose. We present a case of median arcuate ligament syndrome in a 68-year-old woman, whose diagnosis and treatment were delayed by many years as her symptoms were taught to be the result of functional abdominal pain syndrome, ultimately corrected by laparoscopic decompression of the celiac axis. This case demonstrates that surgical decompression of the celiac axis is an effective treatment for median arcuate ligament syndrome and the importance of continuing to reassess the clinical picture of patients labeled with functional abdominal pain syndrome.

## Introduction

The median arcuate ligament is a fibrous structure that connects the right and left crura of the diaphragm. It is located anterior to the aortic hiatus, through which the aorta, azygous vein, and thoracic duct pass. Median arcuate ligament syndrome (MALS) refers to anatomical compression of the celiac artery and/or ganglion by fibrous attachments of the median arcuate ligament [1]. MALS typically presents as a vague constellation of symptoms including anorexia, epigastric/postprandial pain, nausea, and vomiting. As its symptoms closely mimic those of other abdominal pathologies, MALS is often considered a diagnosis of exclusion [2,3]. Surgical decompression can provide effective symptom relief and tends to have superior outcomes compared to conservative management alone [4]. Herein we present a case of MALS masquerading as functional abdominal pain syndrome, before ultimately undergoing laparoscopic decompression. This case is unique in that it demonstrates how MALS can be misdiagnosed if no radiographic evidence of celiac artery compression is found during the initial diagnostic workup.

This article was previously submitted as a meeting abstract to the 2022 American Physician Scientist Association Annual Northeast Regional Conference on January 15, 2022.

## Case presentation

A 68-year-old female, who developed unexplained abdominal pain in the 1990s, was eventually diagnosed with MALS in 2021 after abdominal magnetic resonance angiography (MRA) showed compression of the proximal celiac axis. Her past medical history includes major depressive disorder (managed with a benzodiazepine and selective serotonin re-uptake inhibitor), chronic pain syndrome (managed with opioids and pregabalin), and an extensive gastrointestinal history including peptic ulcer disorder (managed with a proton pump inhibitor), reflux esophagitis, chronic nausea and vomiting (managed with ondansetron), and generalized abdominal pain spanning over 20 years. Her past surgical history includes a total hysterectomy and unknown gastric surgery performed prior to 1990, and numerous orthopedic surgeries.

She initially presented to the gastroenterology (GI) service in the 1990s for unexplained abdominal pain. An unknown workup was performed, and her pain was considered functional. She was not given a definitive diagnosis. She was first seen in our system in 2007. She presented to the Emergency Department (ED) with nausea, vomiting, flank pain, and general abdominal pain. Her workup consisted of abdominal computed tomography (CT) with contrast, urinalysis (UA), complete blood count (CBC), and complete metabolic panel. Abdominal CT showed no abnormal findings, and review of a previous abdominal CT scan showed dilated biliary duct post-cholecystectomy and diverticulosis. Aspartate transaminase (AST), alanine transaminase (ALT), and alkaline phosphate were elevated. Amylase, CBC, lipase, and UA were within normal limits. No definitive diagnoses were provided at that time. Her symptoms were managed with prochlorperazine and oxycodone. She was discharged from the ED and scheduled for an outpatient follow-up with GI the next day. The patient was unable to follow up with GI.

From 2008 onward, she continued to receive care for abdominal pain in inpatient and outpatient settings. Her symptoms were attributed to various causes including irritable bowel syndrome, fibromyalgia, and chronic pancreatitis. She was admitted multiple times for epigastric pain, but her workup continued to remain negative. In 2019, the patient was again admitted due to uncontrolled abdominal pain. An abdominal MRA showed the arcuate ligament compressing the proximal celiac artery. Vascular surgery considered mesenteric ischemia to be unlikely as the superior and inferior mesenteric arteries were widely patent on previous MRA and the patient was often able to tolerate meals without postprandial pain. MALS was acknowledged as a possible diagnosis. The patient was recommended to undergo further evaluation of this; however, the patient did not attend her follow-up appointment.

**Figure 1:**
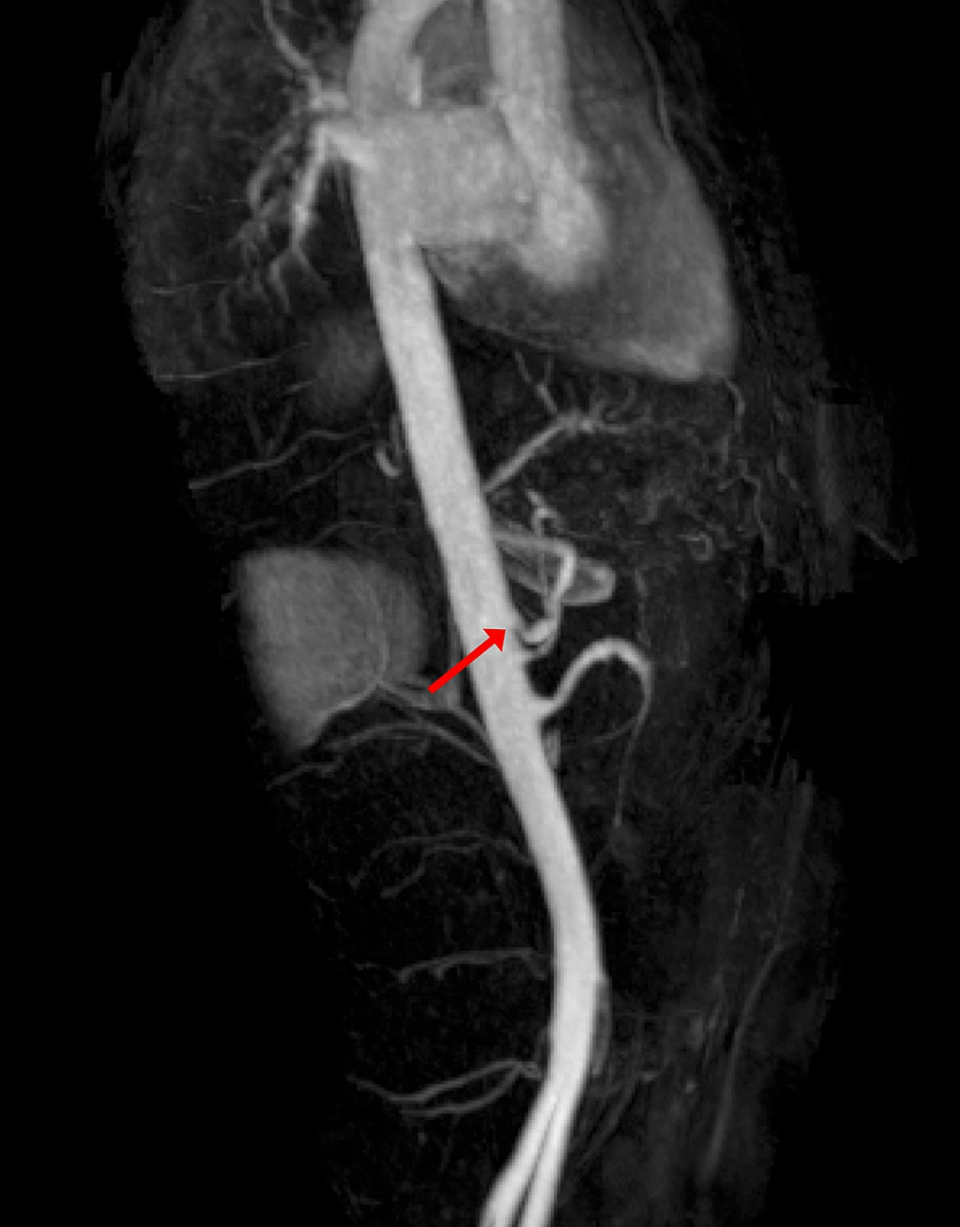
Sagittal maximum intensity projection MRA image demonstrating superior compression of the proximal celiac axis (arrow). MRA, magnetic resonance angiography

**Figure 2:**
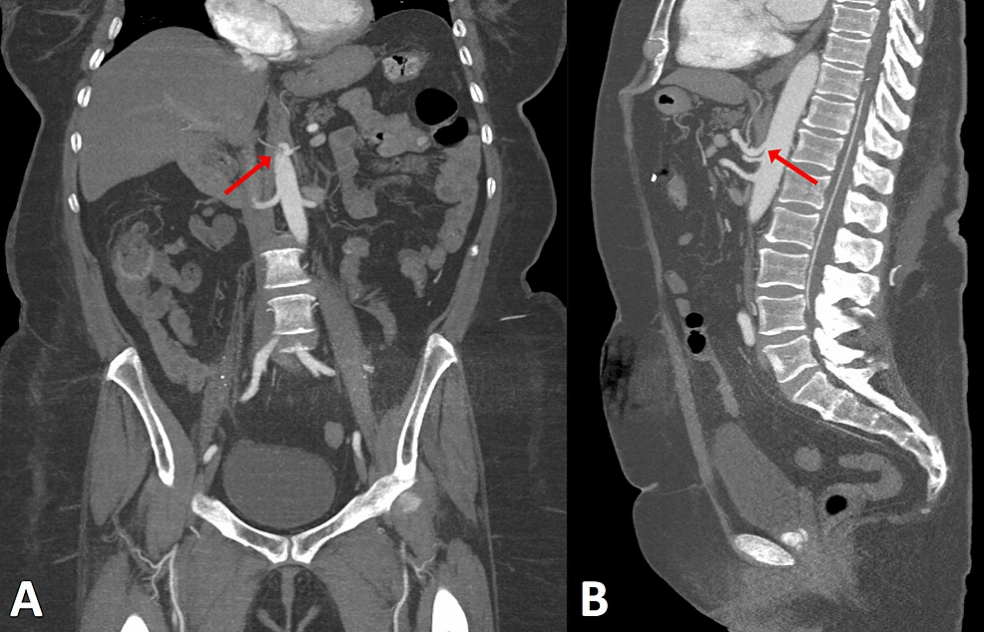
CT imaging of celiac artery stenosis (arrows) in the coronal (A) and sagittal (B) planes. CT, computed tomography

**Figure 3:**
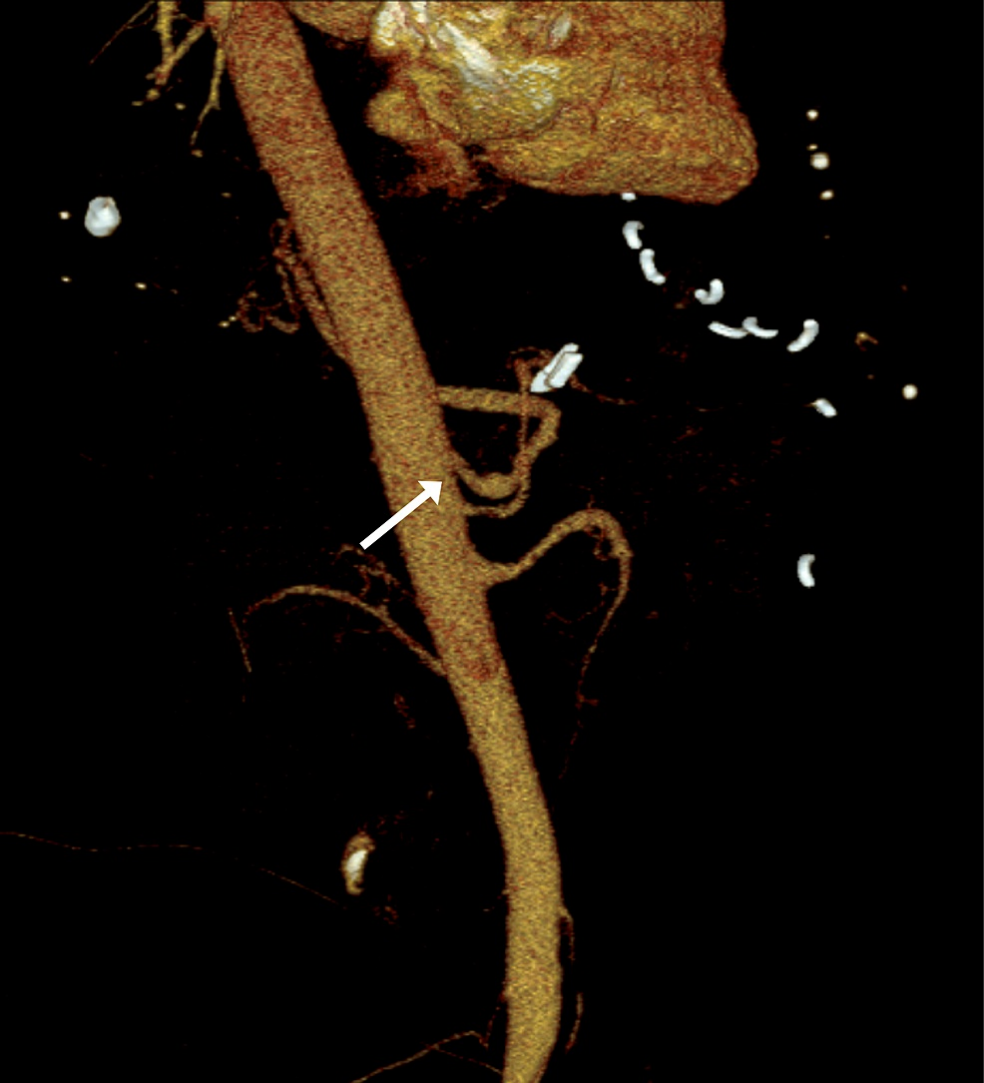
Three-dimensional reconstruction of the aorta showing compression of the celiac artery (arrow).

In 2021, the patient presented to the ED several times for abdominal pain with associated nausea and vomiting, with several admissions for symptomatic management. The patient was counseled on laparoscopic surgical decompression of the celiac trunk; however, she elected not to proceed with this. Two weeks after this, the patient presented with worsened symptoms, and MRA (Figure 1) showed more severe compression of the proximal celiac axis with a strong possibility of MALS. Follow-up CT (Figures 2A, 2B) showed stenosis of the celiac artery near its origin from the aorta and mild post-stenotic dilatation due to a non-obstructing median arcuate ligament. After further discussion, the patient agreed to proceed with laparoscopic lysis of adhesions and release of the median arcuate ligament. A three-dimensional reconstruction of the patient’s aorta was rendered to assist with operative planning (Figure 3).

**Figure 4:**
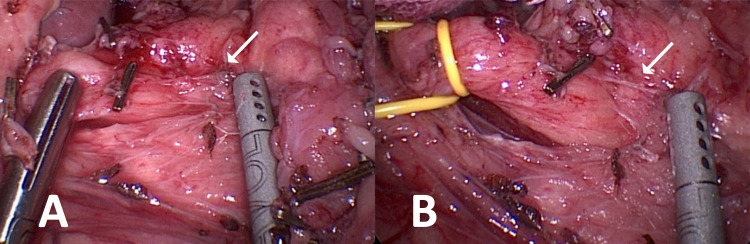
Fibers from the median arcuate ligament (arrows) being dissected off the celiac trunk (A). A Penrose (yellow) was later placed to facilitate retraction (B).

Intra-operatively, several adhesions were noted, and a laparoscopic adhesiolysis was performed. The tissues overlying the celiac trunk were meticulously dissected until the trifurcation and origin of the celiac artery were clearly visible. A thickened band of connective tissue consistent with the median arcuate ligament was seen compressing this area (Figures 4A, 4B). These fibers were divided with laparoscopic scissors until the compression over the celiac artery was fully released.

**Figure 5:**
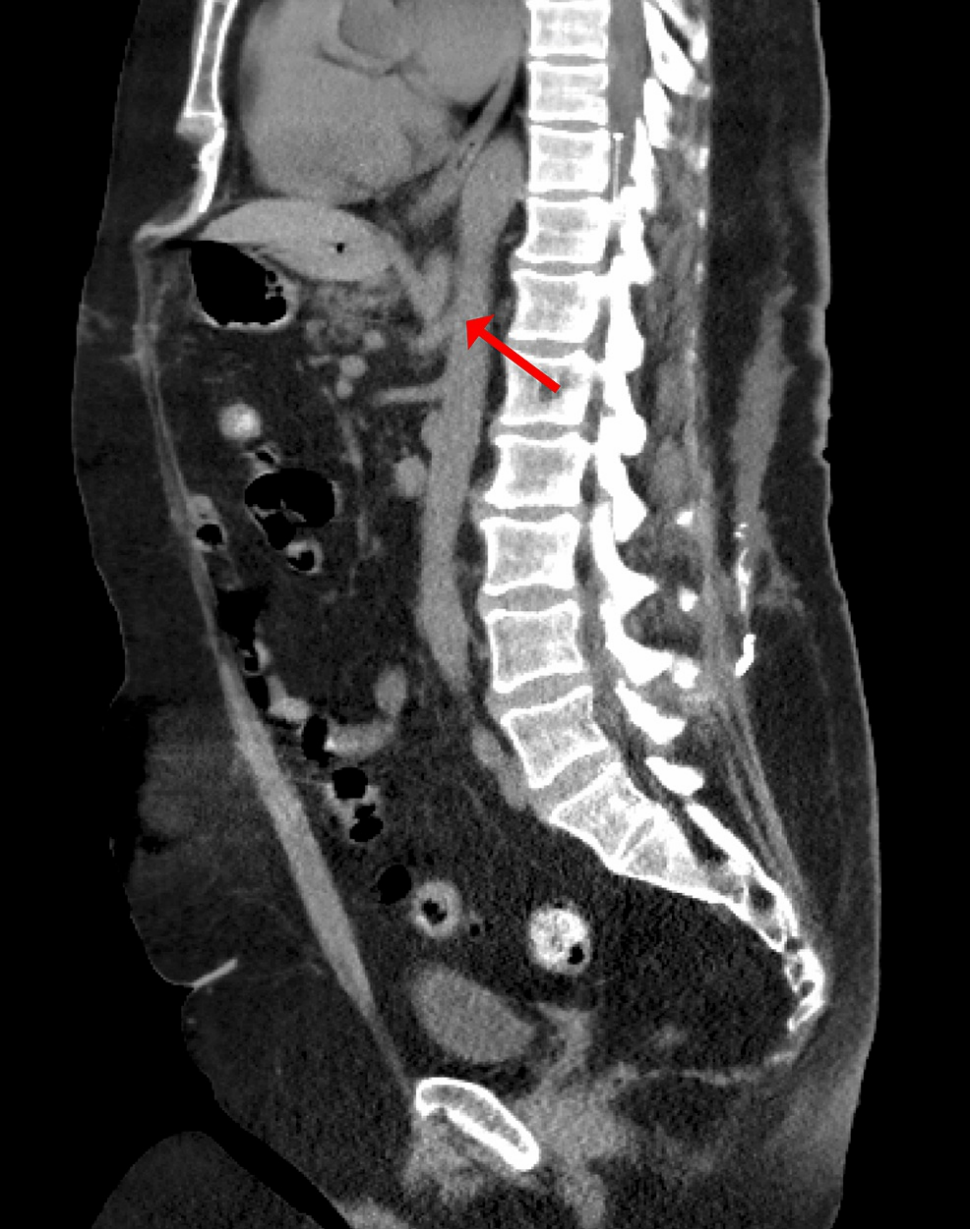
Post-operative imaging of the celiac artery (arrow).

The patient’s post-operative course was relatively unremarkable. She was discharged home a few days post-operatively, and had an outpatient follow-up with both GI and surgery. At five weeks post-surgery, the patient was tolerating a solid diet, had no nausea or vomiting, and had marked improvement in her chronic abdominal pain. A post-operative CT of the abdomen (Figure 5) was obtained two months later, which demonstrated significantly increased patency of the celiac artery.

## Discussion

MALS, also known as Dunbar syndrome or celiac artery compression syndrome, typically presents as a vague constellation of abdominal symptoms [5-7]. In a study of 36 patients with MALS, 94% reported abdominal pain, 80% reported postprandial pain, 56% reported nausea and vomiting, 50% experienced weight loss, and only 8% stated they had abdominal pain triggered by exercise [8]. The etiology of MALS is not clearly understood but is believed to be related to either ischemia or nerve injury secondary to the compression caused by the median arcuate ligament or celiac plexus dysfunction [5]. MALS is a rare condition with an unknown incidence and prevalence. However, it is four times more likely to occur in women than men and is most common in patients between 30 and 50 years of age with a thin body habitus [9]. Symptoms have been found to occur more frequently in patients with more pronounced celiac artery compression on imaging and those with prior abdominal surgery [10]. On physical examination, patients may have abdominal bruits (85%), but up to 30% of patients without this condition may also have this finding [11]. As its symptoms closely mimic those of other abdominal pathologies, MALS is often considered a diagnosis of exclusion. It is typical for patients to be worked up for a variety of gastric, enteric, pancreatic, or sometimes cardiac pathologies before they are ultimately given a diagnosis of MALS. Conventional angiography is considered the gold standard diagnostic method; however, this is an invasive procedure. Compression of the celiac artery happens on end-expiration and is mostly relieved on end-inspiration. It is the relaxation of the diaphragm on expiration that tightens the median arcuate ligament [12]. MRA is performed with random breathing, and the CT scan is typically performed at end-inspiration. Therefore, the compression is more pronounced on MRA as opposed to CT. This explains why our patient’s diagnosis was not identified earlier via CT. Duplex ultrasound and CTA/MRA have been recently shown to be very effective in diagnosing MALS. Diagnostic features on imaging include a peak systolic velocity of >200 cm/s on duplex ultrasound or >50% stenosis of the celiac artery on angiography [7].

The mainstay treatment for MALS is surgical decompression by dividing the fibers compressing the celiac trunk. Open and laparoscopic techniques of median arcuate ligament release have been considered the standard operation, but new robotic approaches have gained popularity in recent years [6]. Some alternative procedures will also remove the celiac plexus [13]. Although operative management is typically superior to non-operative management, long-term follow-up data comparing the two are lacking [4].

Surgical intervention does not always lead to full clinical resolution for patients. Approximately 15-25% of patients can have persistent symptoms with successful surgery [5], although delineation of post-operative pain compared to persistent abdominal pain may take at least one month [14]. This lack of clinical success is generally thought to be related to the lack of criteria available for identifying surgical candidates with the best likelihood of symptom resolution. One study suggested that persistent symptoms should be investigated for a co-morbidity or unresolved stenosis of the artery [6]. An initial surgery that fails to resolve the patient's symptoms may suggest the patient is a candidate for further interventions such as endovascular techniques or vascular bypass [11]. Endovascular stent placement by interventional radiology or vascular surgery has become the preferred treatment in cases of residual stenosis or aneurysm development of the celiac artery following primary surgical decompression. In these cases, endovascular therapy has shown superior outcomes when compared to reoperation. However, endovascular therapy is not recommended as the sole treatment for MALS due to high failure rates [15]. Another study suggested that laparoscopic surgery could be less successful than other techniques because chronic damage to the celiac trunk or fibrosis may be present, although more research is needed [16].

Our case highlights how difficult MALS can be to diagnose. Since presentation our patient has received an extensive workup over a long period of time across multiple subspecialties, a common finding in patients with this rare clinical condition. She was felt to have functional abdominal pain syndrome. This case highlights the importance of reassessing and reevaluating functional abdominal pain syndrome when appropriate.

## Conclusions

MALS is a rare condition that most commonly presents as vague abdominal pain. In this paper, we have presented a case of MALS diagnosed in a patient with chronic abdominal pain felt to be from functional abdominal pain syndrome. When evaluating patients with chronic functional abdominal pain, it is important to continuously evaluate for underlying pathologic causes that can direct definitive treatments, in addition to providing symptomatic management in the interim period. Further clinical research is warranted to better understand the pathophysiology of this disease, its surgical management, and long-term clinical outcomes of this patient population.

## References

[1] Grotemeyer D, Duran M, Iskandar F, Blondin D, Nguyen K, Sandmann W (2009). Median arcuate ligament syndrome: vascular surgical therapy and follow-up of 18 patients. Langenbecks Arch Surg.

[2] Kim EN, Lamb K, Relles D, Moudgill N, DiMuzio PJ, Eisenberg JA (2016). Median arcuate ligament syndrome-review of this rare disease. JAMA Surg.

[3] Ologun GO, Snyder H, Hannigan C, Njoku D, Aman M (2019). Celiac artery compression syndrome in a middle-age woman treated laparoscopically. Cureus.

[4] Goodall R, Langridge B, Onida S, Ellis M, Lane T, Davies AH (2020). Median arcuate ligament syndrome. J Vasc Surg.

[5] Rodriguez JH (2021). Median arcuate ligament syndrome: a clinical dilemma. Cleve Clin J Med.

[6] Fernstrum C, Pryor M, Wright GP, Wolf AM (2020). Robotic surgery for median arcuate ligament syndrome. JSLS.

[7] Ho KK, Walker P, Smithers BM (2017). Outcome predictors in median arcuate ligament syndrome. J Vasc Surg.

[8] Garriboli L, Miccoli T, Damoli I, Rossini R, Sartori CA, Ruffo G, Jannello AM (2020). Hybrid laparoscopic and endovascular treatment for median arcuate ligament syndrome: case report and review of literature. Ann Vasc Surg.

[9] Trinidad-Hernandez M, Keith P, Habib I (2006). Reversible gastroparesis: functional documentation of celiac axis compression syndrome and postoperative improvement. Am Surg.

[10] Khrucharoen U, Juo YY, Sanaiha Y, Finn JP, Jimenez JC, Dutson EP (2020). Factors associated with symptomology of celiac artery compression and outcomes following median arcuate ligament release. Ann Vasc Surg.

[11] Mehta A, Bath AS, Ahmed MU, Kenth S, Kalavakunta JK (2020). An unusual presentation of median arcuate ligament syndrome. Cureus.

[12] Smereczyński A, Kołaczyk K, Kiedrowicz R (2021). New perspective on median arcuate ligament syndrome. Case reports. J Ultrason.

[13] Sun Z, Zhang D, Xu G, Zhang N (2019). Laparoscopic treatment of median arcuate ligament syndrome. Intractable Rare Dis Res.

[14] Kuruvilla A, Murtaza G, Cheema A, Arshad HM (2017). Median arcuate ligament syndrome: it is not always gastritis. J Investig Med High Impact Case Rep.

[15] Tracci MC (2015). Median arcuate ligament compression of the mesenteric vasculature. Tech Vasc Interv Radiol.

[16] San Norberto EM, Romero A, Fidalgo-Domingos LA, García-Saiz I, Taylor J, Vaquero C (2019). Laparoscopic treatment of median arcuate ligament syndrome: a systematic review. Int Angiol.

